# Streaming Support for Data Intensive Cloud-Based Sequence Analysis

**DOI:** 10.1155/2013/791051

**Published:** 2013-04-24

**Authors:** Shadi A. Issa, Romeo Kienzler, Mohamed El-Kalioby, Peter J. Tonellato, Dennis Wall, Rémy Bruggmann, Mohamed Abouelhoda

**Affiliations:** ^1^Center for Informatics Sciences, Nile University, Giza, Egypt; ^2^IBM Innovation Center, Zurich, Switzerland; ^3^Center for Biomedical Informatics, Harvard Medical School, Boston, MA, USA; ^4^Department of Biology, University of Bern, Bern, Switzerland; ^5^Systems and Biomedical Engineering Department, Faculty of Engineering, Cairo University, Giza, Egypt

## Abstract

Cloud computing provides a promising solution to the genomics data deluge problem resulting from the advent of next-generation sequencing (NGS) technology. Based on the concepts of “resources-on-demand” and “pay-as-you-go”, scientists with no or limited infrastructure can have access to scalable and cost-effective computational resources. However, the large size of NGS data causes a significant data transfer latency from the client's site to the cloud, which presents a bottleneck for using cloud computing services. In this paper, we provide a streaming-based scheme to overcome this problem, where the NGS data is processed while being transferred to the cloud. Our scheme targets the wide class of NGS data analysis tasks, where the NGS sequences can be processed independently from one another. We also provide the *elastream* package that supports the use of this scheme with individual analysis programs or with workflow systems. Experiments presented in this paper show that our solution mitigates the effect of data transfer latency and saves both time and cost of computation.

## 1. Introduction

Over the past few years, cloud computing has emerged as a new form of providing scalable computing resources on demand. Customers using cloud services have access to remote computational resources that can be scaled up and down and they are charged according to the time of utilization. The cloud model is appealing for many scientific applications, where large computational resources are required on an ad hoc basis for analyzing large datasets produced or collected after some experimental work. Currently, there are a number of academic as well as commercial cloud computing providers worldwide; these include Amazon Web Services (AWS) [1] (which pioneered the provision of such services), Microsoft Azure [2], IBM Smart Cloud Enterprise [3], Rackspace [4], Magellan [5], and DIAG [6], to name a few.

The bioinformatics academic community has already recognized the advantages of cloud computing since its early days and considered it as a promising solution to overcome the ever increasing genomic data volume [7–12], especially for the scientists with limited computational power. Cloud-based software tools have been developed by the academic community for the analysis of biological sequences. These include, among others, Crossbow [13], RSD-Cloud [14], Myrna [15], and CloudBurst [16]. The life science industry has moved in the same direction and started to support cloud computing as well. Interestingly, recent NGS instruments can stream the sequenced reads to the cloud infrastructure during the sequencing process (https://basespace.illumina.com/). This has the advantage that all the new sequence data become available in the cloud upon completion of the wet-lab work.

This exciting advancement in providing cloud-based bioinformatics services is however limited by the latency of copying the user's data to the computing machines in the cloud. To take one example, uploading the African human genome dataset (130 GB) takes around 37 hours with upload rate of one MB/s, while processing this dataset (as we will show in the experiment) using Bowtie [17] takes about 32 hours. That is, the data transfer time can exceed or at least be a considerable fraction of the processing time. This directly increases the overall experiment time and accordingly increases the associated costs. Current solutions to overcome this problem are mostly commercial and they only focus on the reduction of the data transfer time, by using faster data transfer protocols and compression techniques (c.f., [18], http://www.filecatalyst.com/ and http://asperasoft.com/). These solutions are however limited by the user's bandwidth and the nature of the data that is compressed and transferred. In this paper, we show that it is possible to further reduce the overall experiment time by incorporating an online data processing (streaming) scheme to process the data while it is transferred. This solution fits the wide class of NGS problems, in which the NGS sequences can be processed independently from one another; the problems of mapping NGS sequences to a reference genome or searching them in a given set of databases are examples of these problems. In the aforementioned example of the African human genome, the overall processing time using our scheme will converge to the data transfer time, as the data transfer and computation proceed in parallel. As we will show in the paper, this scheme has the extra advantage of reducing the overall cost of the experiment due to the use of fewer compute nodes.

In this paper, we present the incremental (online) data processing package *elastream* (*elastic-stream*) that has the following set of features:

- automatic creation and management of a computer cluster in the cloud (including MapReduce clusters), equipped with necessary NGS analysis tools,
- automatic submission of jobs to the cluster and monitoring them,
- incremental (online) data processing for individual tools as well as for workflow engines installed on the cloud machines, even if the tools and engines do not directly read/write to standard Unix pipes,
- adaptive load balancing where the number of cluster nodes can be increased or decreased in run time in response to changes in the computation load.

To further facilitate the use of *elastream* for individual applications, we provide a client software that can be used from the user's local machine to activate the *elastream* cloud cluster and submit analysis jobs to it. Furthermore, we also provide add-on's in the form of workflows to enhance the popular workflow systems Taverna [19, 20] and Galaxy [21]. These add-on's facilitate the use of cloud computing power with the data streaming option. These add-on's are useful for the developers and users of the Taverna and Galaxy systems to scale up their resources and enhance the performance of their workflows.

This paper is organized as follows: Section 2 includes related work and a summary of the Amazon cloud computing products. In Section 3, we introduce our *elastream* package, which supports establishment and use of a cloud computing cluster. In this section, we explain the design principles as well as the implementation details of *elastream*.  Section 4 introduces our on-line processing scheme for individual tools as well as for workflow systems.  Section 5 introduces the features of *elastream* distribution and its add-ons. In Section 6, we present a demonstration of our scheme based on *elastream* in the Galaxy workflow system. We also evaluate the performance of the streaming solution. Finally, Section 7 includes the conclusions and future work.

## 2. Background and Related Work

### 2.1. Cloud Computing and Amazon Web Services

#### 2.1.1. Cloud Computing Services

Cloud computing provides access to remote computing resources (processors, memory, storage, bandwidth, software, etc.), where such resources are encapsulated as services that can be metered and charged for on a pay-per-usage basis. From a service-oriented point of view, cloud computing services can be categorized as Infrastructure-as-a-Service (IaaS), Platform-as-a-Service (PaaS), or Software-as-a-Service (SaaS).

The SaaS model provides an abstraction of traditional Internet applications. A piece of software is deployed at the cloud provider's site and is accessed as a remote service. Using this model, analysis tools are deployed as accessible remote services, allowing users to access and execute these tools. Crossbow [13], (http://bowtie-bio.sourceforge.net/crossbow/ui.html), which is hosted at Amazon, is an example of these tools. In this case, users do not need to worry about the low-level issues related to resource allocation and execution of the tool; these are handled by the SaaS provider.

The PaaS model provides an abstraction of a complete development platform deployed as a service. The platform typically comprises a restricted software development environment with an associated software stack. This enables application builders to develop new programs, usually for specified classes of applications. A scientific workflow system, for example, Galaxy [21], deployed in the cloud is an example of a PaaS. In this case, again, it is the PaaS provider who handles all resource allocation decisions and low level details of workflow execution.

Within the IaaS model, a cloud service provider, such as Amazon, hosts large pools of computing machines and offers access to them via a set of APIs. Users can configure the machines as they wish (operating system, software, etc.) and then use them for executing their applications. The machines provided are commonly virtual machines (VMs) that the provider manages on behalf of the consumer. For the provider, the use of the VM abstraction supports scalability by allowing a physical machine to be shared by multiple VMs and also allowing them to bill users only for the time the VMs are running. For the user, any number of virtual machines can be allocated and configured. Moreover, SaaS and PaaS applications can be freely installed and used. In this case, the user has to handle decisions about VM configuration and software installation as well as about allocation and de-allocation of the VMs based on performance and budget requirements.

#### 2.1.2. Amazon Web Services

Because the current version of *elastream* is based on the Amazon cloud infrastructure, we review the basic technical and financial features of this infrastructure. Our use of the Amazon platform is motivated by the fact that it is the largest and most popular one so far. Though, we would like to stress that the methods and approaches presented in this paper are applicable to any cloud computing platforms and are not specific to Amazon; it is planned that future versions of *elastream* will support more platforms. 

Amazon Web Services (AWS) of Amazon offers infrastructure as a service (IaaS) in terms of computational power (CPUs and RAM), storage, and connectivity. AWS offers a variety of machine instance types that range in computing power and cost.  Table 1 summarizes the features of some instance types including the strongest ones. With each of these types, mounted disks (called *ephemeral* disks) are also provided. Machine instances are created from Amazon Machine Images (AMIs), which are templates containing software configurations (e.g., operating system, application server, and applications). AWS includes a directory of AMIs prepared by the AWS team and by the community. The deposited AMIs in this directory have different operating systems and are equipped with different applications.

Because the ephemeral disks are volatile and vanish with the termination of the virtual machine, AWS offers two types of persistent storage: EBS and S3. The former is defined in terms of volumes, where one or more EBS volumes can be attached (mounted) to a running virtual machine instance, similar to a USB thumb drive (volume size ranges between 1 GB and 1 TB). The latter is like a data center providing data hosting, accessed through certain methods (basically POST and GET methods).

The AWS business model is “pay-as-you-go,” where the user is charged only when own machines are running. The user is also charged for reserved storage on Amazon and for data transfer out of the AWS site and from/to persistent storage solutions.  Table 1 summarizes the storage options and their prices in AWS (last price update November 2012). For more information about the AWS pricing schemes, we refer the readers to the documentation available in the AWS website [1]. 

### 2.2. Related Work

#### 2.2.1. Cloud-Based Solutions for Sequence Analysis

Currently, there are some cloud-based programs for the analysis of next-generation sequencing data. These include, among others, Crossbow [13], RSD-Cloud [14], Myrna [15], and CloudBurst [16]. In addition, there are some libraries and packages that support the creation and management of computer clusters in the cloud. To the best of our knowledge, these include so far StarCluster [22], Vappio [23], and CloudMan [24]. StarCluster [22] has been developed as a general cluster management solution for AWS and it is not specific to bioinformatics applications or any bioinformatics use cases. CloudMan [24] has been developed as part of the Galaxy project to basically provide a version of the Galaxy workflow system [21, 25] in the cloud. Vappio [23], unlike CloudMan, is a standalone library for supporting the creation of a computer cluster in the cloud. It enables submission of remote jobs to the cloud instances. These solutions assume that the data should be available in the cloud before any processing takes place. Our work in this paper can be used to enhance these solutions with incremental processing features.

#### 2.2.2. Online Data Processing

Online data processing (also referred to as stream or incremental data processing) has been addressed since the early days of distributed computing, especially in the area of distributed database systems. Specifically, pipelined query evaluation models have been introduced to hide data transfer latencies within the processing of the queries [26]. The same approach can be readily used in other applications and over a computer cluster empowered by a job-scheduler (e.g., PBS) to manage job submissions. Online processing with the MapReduce framework is relatively new and has just appeared in [27, 28]. The involved approach in these papers is based on modifying the MapReduce implementation and providing a stream-based data processing system underneath. The problem of these solutions is that they have not yet been supported by the Amazon MapReduce product. In this paper, we will overcome this limitation by following a different approach based on the elasticity property of the cloud model. But once it is supported by Amazon, we will enhance our package with this feature to further serve the bioinformatics community. 

In preliminary work [29, 30], we evaluated the incremental data processing approach for certain bioinformatics tools like SHRiMP [31] and Bowtie [17] based on an industrial streaming engine (IBM InfoShphere Streams). In this paper, we extend this work in several directions: first, we introduce a scheme that supports stream processing in a generic way with no dependencies on any streaming engine. Second, our scheme is applicable not only to specific software tools but also to workflow systems like Galaxy [21] and Taverna [19, 20]. Finally, our work supports incremental processing over the Elastic MapReduce framework of AWS.

## 3. *Elastream*: Design and Implementation

### 3.1. Block Diagram

**Figure 1:**
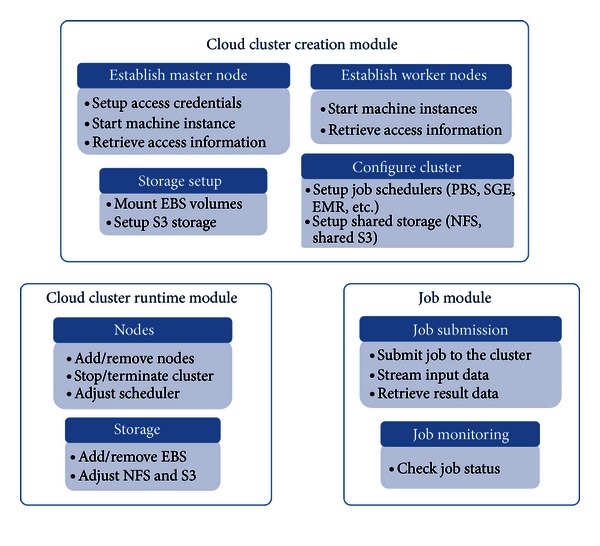
*Elastream* block diagram. *Elastream* is composed of three modules. Each module includes submodules conducting certain tasks.

*Elastream* is a software package composed of a set of modules for constructing a computer cluster in the cloud and executing analysis jobs on it.  Figure 1 shows the block diagram of *elastream*. As shown in Figure 1, the package is composed of three basic modules: *cloud cluster creation module*, *cloud cluster runtime module*, and *job module*.

#### 3.1.1. Cloud Cluster Creation Module

This module includes functions for creation of the cluster in the cloud. These functions can be categorized into three submodules.

The first submodule includes functions for setting up the master node of the cluster. The master node is created from the *elastream* virtual machine image, which we have already prepared and deposited in AWS as AMI. This virtual image includes the Linux operating system and all necessary software libraries and packages. It also includes the whole *elastream* package. (Detailed description of the image is given in Section 5.) This sub-module is based on invoking certain APIs provided by AWS. The activation of the cluster node includes some built in bootstrapping scripts that conduct necessary configuration steps (e.g., SSH key settings) and installation of some important libraries and packages.

The second submodule includes functions for creating the worker nodes and associating them to each other and to the master node. The worker nodes are created from the same *elastream* virtual machine image used for creating the master node. This sub-module also includes installation and configuration of the job scheduler (PBS Torque is the default job scheduler) over the created worker nodes.

The third sub-module includes functions for creating the EBS volumes and attaching them to the cluster nodes. It also includes functions for connecting the nodes to the S3 storage to save the result data. There are also functions to establish shared storage among the cluster nodes using NFS or through S3 using S3fs so that the input data becomes available to every job running at any node. 

#### 3.1.2. Cloud Cluster Run-Time Module

This module includes functions responsible for checking the cluster status and terminating the cluster. It also includes functions for adding more nodes and attaching more EBS volumes and S3 buckets to the cluster nodes. 

#### 3.1.3. Job Module

This module includes functions responsible for submitting jobs to the cluster from a remote machine. It also includes functions for checking job status and redirecting the results to certain directories or to the persistent S3 storage.

### 3.2. Use Case Scenario

**Figure 2:**
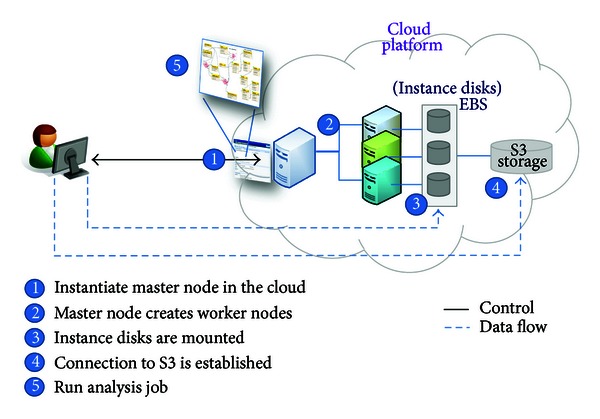
Use case scenario based on the functions of *elastream*. The user uses the client program from own local machine to establish and use a computer cluster in the cloud.

Figure 2 shows the basic use case scenario, in which the functions of *elastream* are used to create a computer cluster in the cloud from remote user's machine and to submit analysis jobs to it. As mentioned before, the cluster is created from the specific *elastream* virtual machine image we have already prepared and deposited in AWS. The first step in this use case scenario is that the user installs the client program of *elastream* from its website. This client program invokes the cluster creation module using the user's credentials so that the created computer cluster is associated with the user's account in AWS. The creation procedure includes the following steps.

First, the function for creating the master node from the *elastream* machine image is invoked. Once the master node is created, a job request is sent to it to execute a program in the master node that creates other worker nodes. This job request includes invocation of the node creation function to create the specified number of worker nodes. Technically, the node creation function is the same as the one used for creating the master node. The only difference is that it already has the credential information, which is reused automatically. We would like to stress that the image of any created machine includes the *elastream* package with all its functions that can be directly used once the machine is activated. After the creation of all worker nodes, another job request is sent to the master node to configure the cluster and the job scheduler. This job invokes a certain script in the cloud cluster to accomplish this task. Once the configuration tasks have been successfully completed, the cluster is ready to run any analysis job.

Running an analysis job can be achieved by using the client program (1) to execute a command line of the analysis tool, (2) to specify the input, and (3) to specify the destination directory of the output. Note that the command line itself can specify that the analysis task runs through the installed job scheduler. Note also that the job should invoke a program already installed in the *elastream* machine. (Note that all *elastream* programs are accessible once the cluster starts.) It is important to mention that this mode of operation does not prevent the user from accessing and utilizing the cluster, using for example the SSH program. The user manual and source code of the *elastream* functions are available on the package website.

### 3.3. Major Implementation Details

#### 3.3.1. *Elastream* Virtual Machine Image

To facilitate the use of *elastream*, we prepared a virtual machine image deposited at Amazon public pages. (The package website includes details about this image and its ID in AWS.) The *elastream* image is based on Ubuntu Linux and it is equipped with a number of software packages, including Amazon Command Line Tools (the APIs of Amazon), PBS Torque as a job scheduler, NFS as a shared file system, s3fs [32] to handle the S3 as a shared file system, Python/Perl interpreters, MPICH2, and C/C++ and JRE. The image comprises a large library of sequence analysis software tools, summarized in the next section. It also contains the ready-to-use *elastream* modules presented above. Furthermore, it includes a server module and a client module to facilitate communication between the nodes as described below.

#### 3.3.2. Client-Server Software Pattern

To facilitate the communication between the local machine and the master node at one side and between the master node and other worker nodes on the other side, we used a client-server software pattern. We developed a server module and preinstalled it in the machine image. This server module starts automatically whenever the respective machine is activated from its image; this includes the master node as well as any worker node. (We use operating system features to enable creation and automatic startup of the server; see the manual for more details.) The server module listens to certain ports, identifies the incoming messages from the client, maps them to one of the functions in the modules discussed above, and executes them. The client connects to the server through the specified port and invokes one of the server functions. Note that the *elastream* machine image includes a copy of the client program so that its functionalities are used inside the cloud to create extra nodes and to submit specific jobs to all or certain worker nodes. The server and client are written in Python, and both of them use APIs of AWS to handle all cloud-related functions. They also use (shell) scripts we developed to configure the cluster and the associated job scheduler. For remote job submissions (from the client program to the cloud cluster), we have developed and used an asynchronous protocol. This protocol is similar to the RESTful protocol and it is implemented in Python.

#### 3.3.3. Establishment of MapReduce Clusters

MapReduce [33] is a programming model and an execution framework that facilitates the processing of large amounts of data on a computer cluster. Amazon offers a product for MapReduce called *Elastic MapReduce* (*EMR*), based on the open source Hadoop implementation of MapReduce.

Compared to job schedulers, the MapReduce model is more complex, as it requires that the analysis task is formulated in terms of a *Map* and a *Reduce* functions. The former function processes the input items in parallel and emits the results as well as some *key-value* pairs. The *Reduce* function uses these pairs to postprocess the output of the *Map* function in parallel. Considerable programming experience is usually needed so as to fit the structure of computation at hand in terms of Map-Reduce functions. Moreover, not all problems can be formulated in the MapReduce model. Nevertheless, the advantage of using the EMR product lies in its lower machine price compared to traditional nodes of the same type (e.g., one c1.xlarge instance costs $0.66 when used in traditional cluster and costs just $0.12 when used in EMR). These reduced costs make it appealing to use the EMR for NGS data processing. Furthermore, various bioinformatics programs are already based on the MapReduce framework and are demonstrated to work using the EMR product. Examples of these tools include Crossbow [13], RSD-Cloud [14], Myrna [15], and CloudBurst [34].

The client program of *elastream* can create an EMR cluster using the APIs of AWS from the user's local machine. The creation steps are similar to that of the traditional cluster but there are some differences due to the MapReduce model and Hadoop implementation. The EMR cluster cannot be created from a user's own image, such as the *elastream* image we prepared. It can only be built from specific EMR images previously created by the Amazon team. The EMR image contains the basic Hadoop code and basic programming languages (Java and Python), but it does not include any analysis software. Therefore, the required analysis programs should be installed using a bootstrap script specified in the creation function. Note that the bootstrap script is executed before the Hadoop system starts, and it can be generally used for any necessary (initialization) tasks related to the required analysis. (The *elastream* manual includes an example of this bootstrap script).

Analysis jobs can be directly submitted, once the cluster is created and Hadoop system starts. The analysis job is specified using a distinct *elastream* command, and it should include the path to the input data as well as the *Map* and *Reduce* functions implemented either in Java or Python. The *elastream* composes a Hadoop job using these items and executes it on the EMR cluster.

#### 3.3.4. Stream Processing Support

To support online data processing, the job submission/execution method of the *elastream* has to be extended with an additional layer. The following section includes the underlying scheme and the implementation details of this layer. In that section, we will discuss this scheme with traditional and EMR clusters. We will also discuss how it can be used within workflow systems.

## 4. Online Sequence Processing

In this part, we describe our method to support on-line sequence processing for both individual analysis tools and workflow systems. Our method does not depend on any streaming engine and does not require that the involved tools or systems are able to read and write to the standard Unix pipes.

### 4.1. Supporting Individual Tools

**Figure 3:**
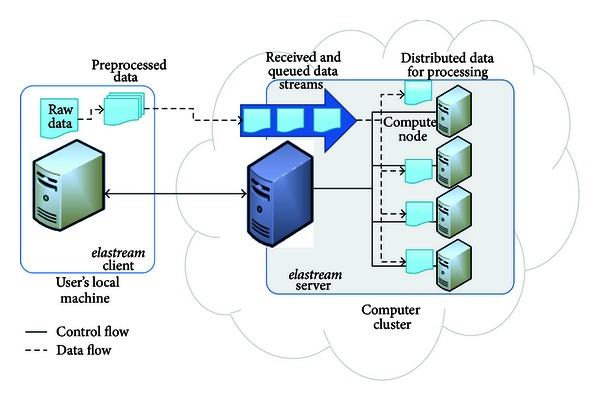
Software design pattern to support incremental data processing in the cloud. The client streams the data into the cloud cluster. The server monitors the received data buckets and manages the launch of analysis jobs on cluster nodes. After completion, the output data is either transferred back to the client machine or transferred to the user's S3 account.

To support incremental data processing in the cloud, we developed the software design pattern shown in Figure 3. This pattern works only for data intensive tasks in which input sequences can be processed independently from one another. Examples of such problems include, among others, mapping NGS sequences to a reference genome and searching sequences in a given set of databases. In this pattern, a local machine streams the data to a cloud cluster and an analysis program already available in that cluster will begin with the processing as soon as the data arrive. To achieve this, there is a server (we call it *streaming server*) installed in the cloud machine and a client program (we call it *streaming client*) at the local machine. The streaming client communicates with the streaming server to start a job in the cloud. The job submission in this pattern includes (1) sending the command line specifying the respective program call and (2) the transfer of the data from the local machine to the cloud machine. Note that the data transfer issue is in sharp what distinguishes this method of job submission from the previousely described offline (nonstreaming based) one, which requires that the input data is completely uploaded to the cloud before starting the analysis. While the data is being transferred, the streaming server monitors the incoming data stream, parses it into sequences, and accumulates the sequences in buckets. When a bucket is complete, the analysis tool is invoked to process the bucket at hand. If the data transfer rate is so high that many buckets are ready at one time point, then more jobs are launched in parallel to process the buckets. After completion, the output data can be downloaded to the user's local machine or exported to an S3 account. 

The client module of this design pattern can do more than establishing a connection to the server and transferring data to it. Actually, it can preprocess the data before sending it to further speed up data transfer and reduce the server side work. This pre-processing includes partitioning the data into chunks and compressing them. In this case, the server expects to receive chunks and it just forward them to the next processing steps.

This design pattern is implemented in *elastream* by extending the functionality of both the server and the client programs. The *elastream* server is extended by two extra threads per job. The first thread is for receiving the data stream, and the second is for monitoring the incoming data and constructing the buckets. The latter thread is also responsible for submitting the jobs to process the completed buckets in a pipelined fashion; that is, a just completed bucket can be directly processed even if the previous buckets are still being processed. The client program can be extended by dividing the data into buckets and sending them in sequence.

### 4.2. Streaming for MapReduce-Based Applications

The *Elastic MapReduce* (EMR) product of AWS does not support incremental data processing and assumes that all the data is available in the cloud in advance. To overcome this limitation, we use the same scheme in which the input data is divided into buckets and these are processed independently. We also make use of the elasticity property of the cloud to expand the cluster when needed. The details are as follows. 

*Elastream* provides a programmatic means for creating and using the *Elastic MapReduce* (EMR) product of AWS. Therefore, an initial EMR cluster is first constructed when an analysis job is submitted. The *streaming server* monitors the received data and accumulates them into buckets. Once a bucket is complete, a Hadoop job is submitted to process this bucket over the EMR. This solution looks fine, but its scalability is in fact limited due to the following reason. EMR is offline in the sense that new buckets cannot join the parallel processing of the currently running job even if there are enough resources, and the new buckets have to be queued to be processed one after another. To overcome this limitation, we exploit the elasticity property of the cloud and automatically create another EMR cluster to process the pending buckets. This elastic creation of EMR clusters enables processing of the buckets with minimal queuing time.

### 4.3. Supporting Workflow Systems

Bioinformatics workflows include the use of multiple software tools and data resources in a staged fashion, with the output of one tool being passed as input to the next. Workflow systems have been introduced to facilitate the design and execution of sophisticated workflows. Examples of the systems that support sequence analysis applications include, among others, Taverna [19, 20], Kepler [35], Triana [36, 37], Galaxy [21], Conveyor [34] Pegasus [38], and Pegasys [39]. In these systems, the workflows are represented in the form of a directed graph, where nodes represent tasks to be executed and edges represent either data flow or execution dependencies between different tasks. The workflow system maps the edges and nodes in the graph to real data and software components. The workflow engine (also called execution or enactment engine) executes the software components either locally on the user's machine or remotely at distributed locations. The engine takes care of data transfer between the nodes and can also exploit the use of high-performance computing architectures so that independent tasks run in parallel. This allows the application scientists to focus on the logic of their applications without worrying about the technical details of invoking the software components or using distributed computing resources. 

**Figure 4:**
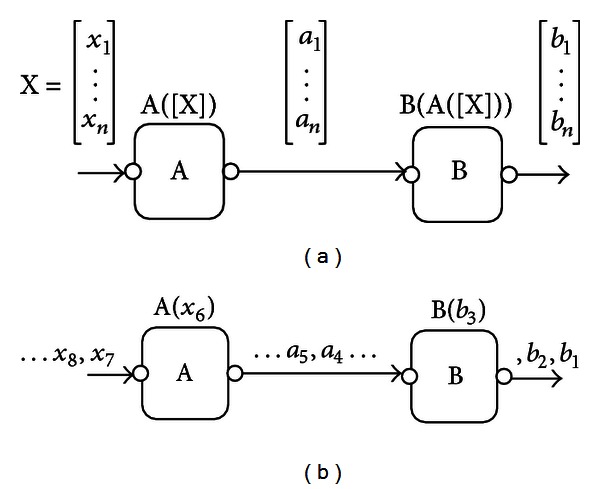
Streaming/pipelining in workflow systems: (a) no pipelining, where A starts computation only when all its input items [*x*_1_ ⋯ *x*_*n*_] are available, and so does B which processes the output items of A. (b) Pipelining, where A starts computation when any data item is available, and so does B. Note that in this mode A and B run concurrently on different data items.

There are two classes of workflow engines: one that supports stream processing (also referred to as *pipelining* in workflow literature) and others that do not. For example, the engines of Kepler [35] and Taverna [19, 20] belong to the first class, while the engines of Galaxy and Conveyor [34] belong to the second one. The idea of pipelining in workflow engines is illustrated in Figure 4. In engines that support pipelining, the output of task A is a list of items [*a*_1_, *a*_2_,…, *a*_*n*_] and task B can start processing whenever an item *a*_*i*_ is produced. That is, tasks A and B can run concurrently in such workflow systems. In engines not supporting pipelining, task A must finish computation over all list items [*a*_1_,…, *a*_*n*_], before B starts processing. 

Changing a workflow system to support pipelining requires some modification of the workflow engine itself, which is a difficult task. Still, this modification is not sufficient per se to achieve efficient online processing during data transfer. This is because some tools within the workflow may not support pipelining at all, which would lead to blocking at some stage of the workflow. To overcome these issues and to support on-line data processing, even for those workflow engines that do not support pipelining, we suggest the following strategy.

We handle the whole workflow system as a usual program that can be invoked from the command line to execute a given workflow over certain input data. In the streaming mode, the streaming server monitors the incoming data items to establish sequence buckets. Once a bucket is ready, the workflow engine is invoked to process this bucket. If multiple buckets are available, multiple instances of the workflow engine are invoked in parallel to process these buckets. This solution permits stream processing even if the workflow engine does not directly support this mode of execution and even if the tools within the workflow do not support pipelining.

## 5. *Elastream* Distribution

### 5.1. Basic Components

The *elastream* distribution includes the package executables, the source code, and the client program to be used from a local machine to invoke package functionality in the cloud. The distribution also includes the following additional features that further facilitate its use for bioinformatics applications.

- We prepared a virtual machine image (AMI) deposited at the AWS machine image directory. The *elastream* package website includes details about this image and its identifier in AWS. This virtual machine image can be used to create a computer cluster from the AWS interface or from our client program installed in the local machine. The *elastream* image includes a set of preinstalled tools that can be directly accessed upon the creation of the cluster. In the current version of *elastream*, there are about 200 tools, coming from BioLinux, EMBOSS [40], SAMtools [41], fastx [42], NCBI BLAST Toolkit [43–45], and other individual sequence analysis programs. Addition of extra tools and updating this image is explained in the *elastream* manual.
- To save costs and to facilitate usage of database-dependent programs, we prepared snapshots of different biological databases and indexes, including the NCBI nucleotide and protein databases in the form of raw and formatted sequences; the raw human genome sequence, and precomputed indexes of it for Bowtie [17]. These snapshots are made available to the user free of charge through a simple interface, to create EBS volumes and mount them to the cluster.

### 5.2. *Elastream* in Workflow Systems

We have developed add-on's (as subworkflows) to support the popular workflow systems Taverna and Galaxy with cloud computing power enhanced with the data streaming option. These add-on's are available as part of *elastream* distribution. 

**Figure 5:**
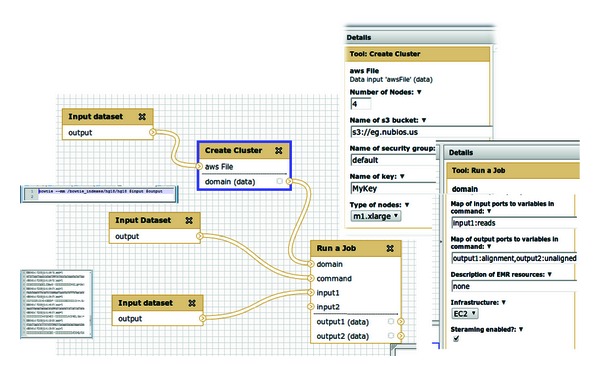
The use of *elastream* in Galaxy in the form of a workflow that establishes the cloud machines and submit jobs. The key nodes of this workflow are the Create Cluster and Run a job. The parameters associated with these nodes are set in the right pane of the web-based interface. We show a part of the file including a command line and a part of another file including NGS sequences; these files are referenced to in the nodes “Input  dataset” connected to the “Run  a  Job.”

For Galaxy, we have created a workflow that enables streaming based on *elastream*. This workflow, which is shown in Figure 5, is composed of the following. There are two major nodes: “Create  Cluster” and “Run  a  Job.” The former is responsible for creating a cluster and the latter is responsible for submitting a job. These two nodes use the functionalities of the *elastream* client program, which is installed within the Galaxy tool set. The *elastream* website includes a link to a Galaxy system with this workflow already built in. The website includes also information for Galaxy administrators on how to integrate this workflow in their systems. We also like to attract the attention that these workflow nodes can be generically used in other user created workflows.

The number and type of machines for the “Create  Cluster” node are specified in an adjacent editing area on the right side of the GUI. The two nodes titled “Input  Dataset” connected to “Create  Cluster” node specify the credential files (certificate and private key files) required to associated the created cluster with the user's account. (Note that input node names cannot be changed in Galaxy).

The “Run  a  Job” node is responsible  for (1) transferring the data from the local user's machine to the cloud machines, (2) invoking a tool (or a system) already installed in the cloud machine, and (3) transferring the result data to the local machines or the S3 cloud storage. The parameters for the “Run  a  Job” node are also specified in an adjacent editing area on the right-hand side of the GUI. The parameters mainly associate the input files to variable names in the command line and specify if streaming is enabled or not. The input node connected to the command port of the node “Run  a  Job” specifies the command line of the analysis program in the cloud. The input node connected to the input1 port specifies the input data. The input to this workflow is a set of sequences to be processed in the cloud. The “Run  a  Job” node includes an optional process to split the input data into chunks before sending them to the cloud.

**Figure 6:**
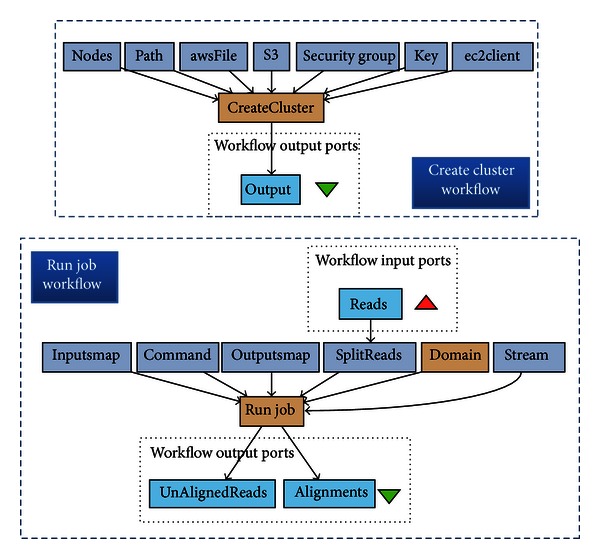
The use of *elastream* with Taverna in the form of a workflow that establishes the cloud machines and submit jobs. The key nodes of this workflow are the Create  Cluster and run  Job nodes. The former is responsible for creating computer cluster in the cloud. The latter workflow submits jobs to the remote cloud machines.

For Taverna, the workflow that enables streaming is composed of two Taverna sub-workflows for management of the cloud cluster and submission of jobs in streaming mode. This workflow can be generically used as sub-workflows in other user created workflows running on Taverna.  Figure 6 shows these two Taverna workflows. The first workflow (called *CreateCluster*) establishes a computer cluster in the cloud. The blue nodes (rectangles) in this workflow define the parameters for creating the cluster. The node titled nodes specifies the number and type of nodes, the node path contains the working directory in the cloud machine, the node awsFile includes the Amazon access credentials, the node S3 states the name of S3 bucket, the nodes securityGroup and key specify the security groups, and the node ec2client defines the path to the *elastream* client tools on the machine where Taverna runs. (Note that the parameters of a node in Taverna are specified through other direct predecessor nodes and not through edit boxes in GUI as in Galaxy.) 

The second workflow (called *runJob*) is responsible for (1) transferring the data from the local user's machine to the cloud machines, (2) invoking a tool (or a system) already installed in the cloud machine, and (3) sending the result data to the local machines or cloud S3. The input to this workflow is a set of sequences to be processed in the cloud. The blue nodes specify the parameters of this workflow: The node titled stream specifies the streaming option, the node command includes the command line to be invoked, and the nodes Inputsmap and OutputsMap map the input and output ports to the command (see the manual for more details). If output ports are not specified, then the result data will not be transferred back to the local machine and remain in the cloud. This workflow includes an optional process to split the input data into chunks before sending them to the cloud.

## 6. Demonstrations and Experiments

In this section, we demonstrate the use of our streaming scheme *elastream* and evaluate its performance. In the following subsection, we demonstrate the use of the Galaxy workflow that supports streaming as discussed in Section 5.2. In the subsequent sub-section, we evaluate the performance of our solution using traditional and EMR computer clusters.

### 6.1. Streaming Cloud-Based Workflows of Galaxy

**Figure 7:**
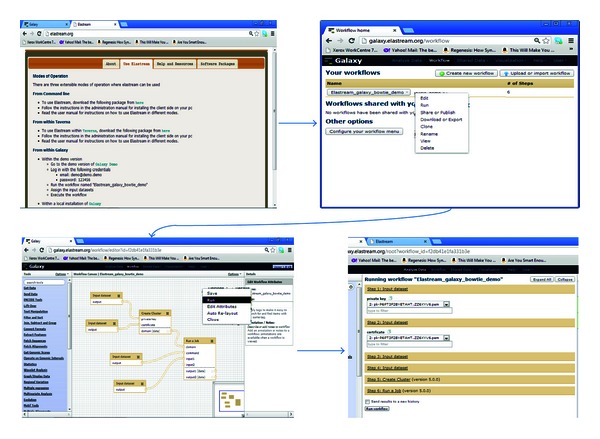
The Galaxy workflow system supported with cloud computing functionalities and streaming option. The upper left screen shot is the *elastream* page, from which the user can access the Galaxy workflow system installed on the *elastream* infrastructure. The upper right screen shot is the Galaxy page, where the user selects the *elastream* workflow. The lower left is the *elastream* workflow, and the lower right is the input/execution page, where the user specifies the paths of the input files including command line, credentials, and input data.

Figure 7 shows the sequence of steps for using the Galaxy workflows, which we have provided to support the use of cloud computing enhanced with the streaming option within Galaxy. From the *elastream* website, the user can access the Galaxy workflow system, which is running on our local infrastructure. Our workflows that support cloud usage with streaming are accessed by selecting the elastream_demo from the workflows drop-down menu. The workflow editing page shows the workflow nodes, where the user can specify the number and type of the cluster machines. If anyone decides to execute this workflow, one will be forwarded to the execution and input page to enter the paths to the credential file, security file, command line file, and input NGS data. For this demo, we have already provided example files for these input types. The example command line includes invocation of the Bowtie program [17] with a set of NGS sequences as input. We stress that this workflow can be used in other Galaxy workflows or with tools other than Bowtie.

### 6.2. Evaluating Performance

We compare two use case scenarios: in the first, the data is processed after it is transferred completely to the cloud (i.e., without on-line processing). In the second, the data is processed while it is transferred (i.e., with on-line processing).

The data intensive task we used in our experiments is the NGS read mapping, where millions of reads have to be aligned to a reference genome. On the traditional cluster, we used the popular program Bowtie [17] as an example tool that performs this task. On the MapReduce cluster, we used the popular Crossbow [13] program. Crossbow is a MapReduce based version of Bowtie implemented using Hadoop and it is enhanced with more functions for SNP detection using soapSNP.

The parameters of these experiments are the data size, the upload speed, and the size of the computer cluster. We used datasets of different sizes, as described in Table 2. The upload speed has been controlled using the program Trickle [46]. To reduce the effects of location, time-of-the-day, and congestion on data transfer, we ran the whole experiments within the Amazon cloud environment. That is, we created one machine to represent the user's machine. This machine is equipped with the Trickle program and the *elastream* client program. The bandwidth we observed within the Amazon site is approximately 20 MB/s to 30 MB/s, which is large enough for our experiments. 

Table 3 shows the runtimes for executing Bowtie in streaming and non-streaming modes using computer clusters of variable size. Each cluster node is of the type c1.xlarge and is composed of 8 cores. Each row in the table shows the upload speed, the upload time, and the computation time for certain cluster size. It also includes the total experiment time without streaming (which is the summation of upload and computation times) and total time with streaming.

From the results in Table 3, we observe the following.

- The use of more machines leads to further reduction of the runtime. For example, it takes 950 minutes to analyze the 130 G human dataset using a cluster of two nodes, and it takes 160 minutes if the cluster size is increased to 16 nodes.
- The streaming mode reduces the overall experiment time, because there is an overlap between data transfer and computation. For example, it takes 508 minutes to upload the 130 G human datasets and to analyze it using a cluster of 8 nodes without streaming (with upload speed of 10 MB/s). With streaming, it takes 320 minutes, which saves about 188 minutes (i.e., *≈*37%). With 16 nodes, it takes 384 minutes without streaming and 235 with streaming (i.e., 38%). One can easily note that the overall experiment time with streaming converges to the overall data transfer time of 224 minutes.
- Comparing the different datasets, we note that the advantage of using on-line processing is more apparent with larger data sizes. For small datasets, like the *E. coli*, where the computation time is neglected, the overhead associated with processing the buckets outweighs the advantage of streaming.
- With slower transfer rate, there is no advantage in using more machines, because there is no much data to be processed in parallel. The* E. coli* and 1 M human genome cases with transfer rates of 250 KB/s and 1 MB/s represent this situation.
- The advantage with respect to the cost of the experiment can be observed when we fix the computation time and compare the experiment cost. For the 130 GB human dataset, the cost of finishing the analysis in 320 minutes using streaming over a cluster with 8 nodes is $31.68. To finish the experiment in the same time, a larger cluster of more than 16 nodes is needed with a cost larger than $34.32.

Table 4 shows the runtimes in minutes for mapping NGS reads to a reference genome using Crossbow based on the Elastic MapReduce (EMR) product of Amazon. In Table 4, there are more than one EMR clusters in each experiment; this is because we establish a new cluster when more buckets become available. The column titled cluster includes the number of these clusters. Each cluster is composed of 4 nodes of the type c1.xlarge. 

In this experiment, the results are analogous to that obtained with the traditional cluster running Bowtie. Here, we also observe that the streaming mode is also superior to the nonstreaming mode with larger datasets. The use of streaming option is not advantageous for small datasets, because Crossbow has some overhead time to preprocess each bucket received, and this overhead outweighs the gain of streaming.

## 7. Conclusions

In this paper, we have introduced *elastream* as a framework for supporting incremental data processing in the cloud. This framework, which is based on the client-server model, is composed of (1) a module for creating and management of cloud computing infrastructure, (2) a module for submission of jobs to the cloud machines with incremental processing feature, (3) a prepared virtual machine image equipped with a large library of bioinformatics tools and databases and all necessary tools, and (4) add-ons in the form of workflows for the popular workflow systems Taverna and Galaxy. 

*Elastream* targets the class of tasks where the input data is composed of large number of sequences that can be processed in parallel. Examples of such tasks include NGS read mapping and blast based queries. Our experiments have shown that the streaming option is useful when the dataset is large enough and the amount of computation at the server size is considerable. With streaming option, one can use fewer machines to finish computation, which leads to reduction of the cost. To sum up, our *elastream* facilitates the use of cloud computing resources for these tasks and its streaming option is of significant advantage to mitigate the effect of the associated data transfer latency. 

Currently *elastream* is limited to AWS and to Linux environment. In future versions, we will extend it to include other cloud providers and the Windows operating system. Streaming for MapReduce requires that the tool has short pre-processing time, as this forms an overhead that limits the use of on-line processing option. In this version of *elastream*, we did not use streaming-based MapReduce solution, because they are not yet supported by Amazon. Once they are supported, we will integrate them as part of our package distribution.

All resources related to *elastream* are available at http://www.nubios.nileu.edu.eg/tools/elastream and http://www.elastream.org/.

## Figures and Tables

**Table 1:** Amazon services: virtual machines, storage, data transfer, and disk access. This information is for the Amazon US site. Prices for other sites are available on the AWS website.

Resource type	AWS service	Service unit	CPUs (#(GHz))	Memory (GB)	Cost ($/Hr)
		m1.large	2 (2)	7.5	0.32
		m1.xlarge	4 (2)	15	0.64
Computation	EC2	c1.xlarge	8 (2.5)	7	0.66
		m2.4xlarge	8 (3.25)	68.4	1.80
		cc1.4xlarge	8 (4.19)	23	1.30

Resource type	AWS service	Service unit	Size	Tiers	Cost ($/GB/month)

	S3	Bucket	Unlimited	1st 1 TB	0.14
Storage	S3	Bucket	Unlimited	Next 450 TB	0.1
	S3	Bucket	Unlimited	Next 4000 TB	0.08
	EBS	Volume	Up to 1 TB		0.10

Resource type	AWS service	Service unit	Type	Size	Cost ($/GB/month)

	S3	I/O	Data IN/within AWS	Any	0.00
	S3	I/O	Data OUT	1st 1 GB	0.00
Data transfer	S3	I/O	Data OUT	Next 10 TB	0.12
	S3	I/O	Data OUT	Next 100 TB	0.07
	S3	I/O	Data OUT	Next 150+ TB	0.05

	S3	API	GET, PUT, POST	1 K request	0.01
Disk access	S3	API	COPY, LIST	1 K request	0.01
	EBS	I/O	R/W	1 M request	0.1

**Table 2:** Description of the used datasets. All the datasets are NGS sequences (reads). The third column includes the number of sequences in millions, and the third column is the data size in GB. There are four sets of the African human genomes of different sizes. The final dataset (130 G) is the original complete one. The three previous ones (1 G, 10 G, 40 G) are subsets of it.

Description	Source	No_Seq	Size
*E. coli* genome	[13]	*≈*9 million	*≈*1.4 GB
1 G African human genome	http://trace.ddbj.nig.ac.jp/dra/index_e.shtml (study SRP000239)	*≈*7 million	*≈*1 GB
10 G African human genome	http://trace.ddbj.nig.ac.jp/dra/index_e.shtml (study SRP000239)	*≈*72 million	*≈*10 GB
40 G African human genome	http://trace.ddbj.nig.ac.jp/dra/index_e.shtml (study SRP000239)	*≈*437 million	*≈*40
130 G African human genome	http://trace.ddbj.nig.ac.jp/dra/index_e.shtml (study SRP000239)	*≈*1419 million	*≈*130 GB

**Table 3:** Running times in minutes for mapping NGS reads to a reference genome using Bowtie based on the use of traditional computer cluster. The column titled upload_speed specifies the upload speed. The column titled “upload” includes the time in minutes for uploading the data to the cloud with the respective upload speed. The column titled “compTime” includes the computation time in minutes of the whole dataset after being uploaded to the cloud. The column titled totalTimeS includes the experiment time in streaming mode and the column titled totalTimeT includes the time in nonstreaming mode, where all the data is first transferred and then processed. The numbers in brackets in this column are the respective monetary cost.

Upload_speed	Read size	Nodes	Upload	CompTime	TotalTimeT	TotalTimeS
*E. coli* reads						

250 KB/s	1.4 G	1	100	3	103 ($1.32)	102 ($1.32)
250 KB/s	1.4 G	2	100	3	103 ($1.98)	102 ($2.64 )
250 KB/s	1.4 G	4	100	3	103 ($3.3)	102 ($5.28)

1 GB human reads						

250 KB/s	1 G	1	71	17	88 ($1.32)	72 ($1.32)
250 KB/s	1 G	2	71	11	82 ($1.98)	72 ($2.64)
250 KB/s	1 G	4	71	7	73 ($3.3)	72 ($5.28)

10 GB human reads						

250 KB/s	10 G	1	800 (13.3 h)	220	1021 ($11.88)	832 ($9.24)
250 KB/s	10 G	2	800 (13.3 h)	130	930 ($12.54)	818 ($9.24)
250 KB/s	10 G	4	800 (13.3 h)	60	860 ($11.88)	818 ($9.24)

*E. coli* reads						

1 MB/s	1.4 G	1	25	3	28 ($0.66)	27 ($0.66)
1 MB/s	1.4 G	2	25	3	28 ($1.32)	27 ($1.32)
1 MB/s	1.4 G	4	25	3	28 ($2.64)	27 ($2.64)

1 GB human reads						

1 MB/s	1 G	1	18	17	35 ($0.66)	21 ($0.66)
1 MB/s	1 G	2	18	11	29 ($1.32)	21 ($1.32)
1 MB/s	1 G	4	18	7	25 ($2.64)	21 ($2.64)

10 GB human reads						

1 MB/s	10 G	1	200	220	421 ($5.28)	231 ($2.64)
1 MB/s	10 G	2	200	130	330 ($5.94)	215 ($5.28)
1 MB/s	10 G	4	200	60	261 ($5.28)	215 ($10.56)

40 GB human reads						

1 MB/s	40 G	1	690	590	1280 ($14.52)	1100 ($12.54)
1 MB/s	40 G	2	690	325	1015 ($15.18)	695 ($15.84)
1 MB/s	40 G	4	690	180	870 ($15.84)	695 ($31.68)

130 GB human reads						

1 MB/s	130 G	1	2220	1720	3940 ($43.56)	3600 ($39.6)
1 MB/s	130 G	2	2220	940	3160 ($45.54)	2400 ($52.8)
1 MB/s	130 G	4	2220	520	2740 ($48.18)	2400 ($105.6)
1 MB/s	130 G	8	2220	284	2504 ($50.82)	2400 ($211.2)

*E. coli* reads						

10 MB/s	1.4 G	1	2.5	3	5.5 ($0.66)	5 ($0.66)
10 MB/s	1.4 G	2	2.5	3	5.5 ($1.32)	5 ($1.32)
10 MB/s	1.4 G	4	2.5	3	5.5 ($2.64)	5 ($2.64)

10 GB human reads						

10 MB/s	10 G	1	18	220	238 ($2.64)	180 ($1.98)
10 MB/s	10 G	2	18	130	148 ($3.96)	85 ($2.64)
10 MB/s	10 G	4	18	60	78 ($3.3)	50 ($2.64)

40 GB human reads						

10 MB/s	40 G	1	70	590	660 ($7.26)	686 ($7.92)
10 MB/s	40 G	2	70	310	380 ($8.58)	350 ($7.92)
10 MB/s	40 G	4	70	170	240 ($8.58)	180 ($7.92)
10 MB/s	40 G	8	70	95	165 ($11.22)	100 ($10.56)
10 MB/s	40 G	16	70	53	123 ($11.88)	73 ($21.12)

130 GB human reads						

10 MB/s	130 G	1	224	1720	1944 ($21.78)	2050 ($23.1)
10 MB/s	130 G	2	224	950	1174 ($23.76)	1100 ($25.08)
10 MB/s	130 G	4	224	520	744 ($26.4)	580 ($26.4)
10 MB/s	130 G	8	224	284	508 ($33.66)	320 ($31.68)
10 MB/s	130 G	16	224	160	384 ($34.32)	235 ($42.24)

**Table 4:** Running times in minutes for mapping NGS reads to a reference genome using Crossbow. The column titled speed specifies the upload speed. The column titled cluster includes the number of created EMR clusters. The column titled compTime incudes the computation time of the whole data after uploading it. The column titled totalTimeS includes the experiment time in streaming mode, and the column titled totalTimeT includes the time in nonstreaming mode, where all the data is first transferred and then processed. The number in brackets in this column is the respective monetary cost.

Upload_speed	Read_size	Clusters	Upload_time	CompTime	TotalTimeT	TotalTimeS
*E. coli* reads						

250 KB/s	1.4 G	1	100	9	109 ($0.72)	110 ($0.96)
250 KB/s	1.4 G	2	100	7	107 ($1.2)	110 ($1.92)
250 KB/s	1.4 G	4	100	7	107 ($2.26)	110 ($3.84)

1 GB human reads						

250 KB/s	1 G	1	71	6	77 ($0.72)	84 ($0.96)
250 KB/s	1 G	2	71	5	76 ($1.2)	84 ($1.92)
250 KB/s	1 G	4	71	5	76 ($1.2)	84 ($3.84)

10 GB human reads						

250 KB/s	10 G	1	800	60	860 ($2.16)	820 ($6.82)
250 KB/s	10 G	2	800	31	831 ($2.64)	820 ($13.44)
250 KB/s	10 G	4	800	18	818 ($3.6)	820 ($26.88)

*E. coli* reads						

1 MB/s	1.4 G	1	25	9	34 ($0.6)	65 ($0.96)
1 MB/s	1.4 G	2	25	7	32 ($1.08)	65 ($1.92)
1 MB/s	1.4 G	4	25	7	32 ($2.04)	65 ($3.84)

1 GB human reads						

1 MB/s	1 G	1	18	6	24 ($0.6)	31 ($0.48)
1 MB/s	1 G	2	18	5	23 ($1.09)	32 ($0.96)
1 MB/s	1 G	4	18	5	23 ($2.04)	31 ($0.1.92)

10 GB human reads						

1 MB/s	10 G	1	200	60	260 ($0.96)	220 ($1.92)
1 MB/s	10 G	2	200	31	231 ($1.54)	220 ($3.84)
1 MB/s	10 G	4	200	18	218 ($2.40)	220 ($7.68)

40 GB human reads						

1 MB/s	40 G	1	690	580	1270 ($6.24)	630 ($5.28)
1 MB/s	40 G	2	690	300	990 ($6.24)	630 ($10.56)
1 MB/s	40 G	4	690	150	840 ($7.2)	630 ($21.12)
1 MB/s	40 G	8	690	80	770 ($9.12)	630 ($42.24)

130 GB human reads						

1 MB/s	130 G	1	2220	1890	4110 ($19.8)	2360 ($19.2)
1 MB/s	130 G	2	2220	945	3165 ($19.8)	2320 ($37.44)
1 MB/s	130 G	4	2220	476	2696 ($19.8)	2320 ($74.88)
1 MB/s	130 G	8	2220	184	2404 ($19.8)	2320 ($149.76)
1 MB/s	130 G	16	2220	126	2346 ($27.48)	2320 ($299.52)

10 GB human reads						

10 MB/s	10 G	1	20	60	80 ($1.08)	160 ($1.44)
10 MB/s	10 G	2	20	31	51 ($1.08)	95 ($1.92)
10 MB/s	10 G	4	20	18	38 ($2.04)	65 ($3.84)

40 GB human reads						

10 MB/s	40 G	1	70	580	650 ($5.04)	610 ($5.28)
10 MB/s	40 G	2	70	300	370 ($5.04)	310 ($5.76)
10 MB/s	40 G	4	70	150	220 ($6.00)	170 ($5.76)
10 MB/s	40 G	8	70	80	150 ($7.92)	110 ($7.68)

130 GB human reads						

10 MB/s	130 G	1	224	1890	2114 ($15.84)	1960 ($15.84)
10 MB/s	130 G	2	224	945	1169 ($15.84)	1000 ($16.32)
10 MB/s	130 G	4	224	476	700 ($15.84)	520 ($17.28)
10 MB/s	130 G	8	224	184	470 ($15.84)	300 ($19.2)
10 MB/s	130 G	16	224	126	350 ($23.52)	300 ($38.4)
